# ‘Odds Are: They Win’: a disruptive messaging innovation for challenging harmful products and practices of the gambling industry

**DOI:** 10.1016/j.puhe.2023.08.009

**Published:** 2023-11

**Authors:** T. Mills, J. Grimes, E. Caddick, C.L. Jenkins, J. Evans, A. Moss, J. Wills, S. Sykes

**Affiliations:** a PHIRST South Bank, London South Bank University, 103 Borough Rd, London SE1 0AA, UK; b Gambling with Lives – The Circle, 33 Rockingham Lane, Sheffield S1 4FW, UK; c Greater Manchester Combined Authority – Tootal, 56 Oxford St, Manchester M1 6EU, UK

**Keywords:** Gambling harms, Gambling taglines, Commercial determinants, Disruptive innovation, Lived experience, Coproduction

## Abstract

**Objective:**

This paper presents an evidence informed rationale for focussing on harmful gambling products and industry practices in public health messaging through the example of a recent innovation called ‘Odds Are: They Win’.

**Methods:**

‘Odds Are: They Win’ was initially developed through coproduction involving public health professionals and people with lived experience of gambling harms and implemented across a city-region area. A review of relevant evidence was undertaken, upon which the research team reflected to draw out the implications of ‘Odds Are: They Win’ for gambling harms messaging.

**Results:**

Evidence is mounting that safer gambling campaigns framed in terms of individual responsibility are ineffective and can generate stigma. ‘Odds Are: They Win’ presents an alternative focus that is not anti-gambling but raises awareness of industry manipulation of the situational and structural context of gambling. This is in-keeping with historical lessons from the stop smoking field and emerging research in critical health literacy. The latter highlights the potential of education on the social and commercial determinants of health to stimulate behaviour change and collective action.

**Conclusion:**

‘Odds Are: They Win’ is a potentially disruptive innovation for the gambling harms field. Research is required to robustly evaluate this intervention across diverse criteria, target audiences, and delivery settings.

## Introduction

Research is increasingly showing that ‘safer gambling’ campaigns, such as ‘Take Time to Think’, are ineffective at stimulating behaviour change,^1^^,^^2^ with many commentators urging a rethink of the contents and aims of such campaigns.^3^, ^4^, ^5^, ^6^ Prior ‘responsible gambling’ campaigns were strongly criticised, from a public health perspective, for contributing to the normalisation of harmful commodity consumption.^7^, ^8^, ^9^ Some public health researchers have highlighted the ambiguous nature of campaign contents, which, they argued, may be strategic, aiming to promote favourable attitudes towards products or the industry rather than educate on harm.^7^^,^^10^ By framing gambling harms in terms of individual responsibility, the gambling industry's role in facilitating harm, through the development and marketing of harmful products, was silenced; responsibility for harm was, instead, pushed onto consumers.^7^, ^8^, ^9^ At the same time, calls for people to gamble responsibly implied that harms are a matter of individual choice or personality type. A binary distinction was constructed between many people who gamble responsibly and those who do not, who are considered to be deficient or faulty in some way.^11^ This can have various consequences, including a tendency to stereotype people who encounter harm (e.g. ‘the problem gambler’), and social stigma.^3^^,^^12^^,^^13^

Gambling harms campaigners and public health researchers have observed how safer gambling campaigns that attempt to move beyond individual responsibility share weaknesses of responsible gambling campaigns.^3^^,^^5^^,^^11^^,^^14^ While some responsibility is afforded to gambling operators to enable safer gambling, this is through self-regulation rather than public policy. Furthermore, a similar binary between people who gamble ‘safely’ and small numbers of vulnerable people is posited, serving to retain much of the responsible gambling framework.^14^

Recent public health commentary on safer gambling campaign taglines has emphasised the practical challenges of shifting conclusively from a focus on individual responsibility. The Australian government has replaced its responsible gambling messages with a set of seven taglines, including ‘Chances are you're about to lose’, designed to avoid generating stigma.^15^ As Marko et al. note^3^, this safer gambling campaign continues to frame gambling harms in terms of individual decisions and behaviours. Highlighting historical lessons from counter-industry campaigns in the tobacco field,^16^ Marko et al. suggest that a focus on harmful products may be more effective at prompting behaviour change and have positive implications for social stigma:^3^ such product- or industry-oriented educational campaigns have, however, been slow to emerge in the gambling harms field.

Here, we review the evidence for shifting away from individual responsibility framings and present a potentially disruptive innovation, called ‘Odds Are: They Win’. Disruptive innovations are those that upend conventional ways of thinking, often starting small before displacing established products and services.^17^ ‘Odds Are: They Win’ disruptively breaks from individual responsibility by focussing on harmful industry products and practices, pointing to new avenues for safer gambling campaigns that, we argue, demand further research.

## Why shift away from individual responsibility?

Health messaging campaigns framed in terms of individual responsibility require an urgent rethink. While some evidence suggests that such campaigns can prompt behaviour change in certain contexts,^18^ experimental studies in gambling frequently find null effects,^1^^,^^2^^,^^19^^,^^20^ and studies warn of considerable unintended consequences. In environments that are already saturated with product marketing, promoting responsible consumption may add further cues to consume with some experimental studies, in both the gambling and alcohol harms fields, suggesting that they can increase use of harmful commodity items.^20^^,^^21^ We have already noted that the binary between responsible/controlled gambling and irresponsible/uncontrolled gambling, which underpins responsible and safer gambling campaigns (see earlier), has been linked to stereotypes and social stigma. People who experience gambling harms appear to internalise this binary such that they view themselves, rather than harmful products, as the source of harm.^3^^,^^12^^,^^13^ This is significant because stigma is a major barrier to help-seeking behaviour.^22^ Such binary assumptions may also impact on how individuals who gamble below high-risk thresholds understand their gambling and the harms they experience. Indeed, recent advances in the alcohol harms field warn that binary assumptions in lay understandings of addiction can impede individual problem recognition, even among individuals experiencing considerable harm from their drinking.^23^ This is partly due to ‘the alcoholic’ stereotype, which constitutes an identity threat that alcohol users distance themselves from.^24^

It may be tempting to conclude, given these limitations, that health messaging campaigns should have no role in public health strategy for gambling harms. Yet, recent developments in the field of critical health literacy, as well as historical examples of counter-industry campaigns in the stop smoking field, suggest that an industry-oriented focus may be more effective at prompting reflection and behaviour change. Social marketing campaigns focusing on cigarettes as the source of harm, when implemented as part of a joined-up public health strategy, have been found to contribute to reduced smoking prevalence.^16^^,^^25^ More recently, public health-framed nutrition interventions have successfully harnessed young people's desire for autonomy and social justice to promote healthy eating through enhanced awareness of manipulative marketing strategies.^26^^,^^27^ Besides these positive impacts on consumption patterns, moreover, an important consideration is the role of counter-industry education in driving social and political change. Recent studies suggest that education on the social and commercial determinants of health may stimulate collective action^28^^,^^29^ and secure public support for public health policies, in part by inoculating consumers from industry misinformation.^30^ The question, then, is not whether we should invest resources in educational, health messaging campaigns but rather what form such campaigns should take.

## Why focus on the gambling industry?

The Betting and Gambling Council (BGC), which represents the gambling industry in the UK, frequently cite prevalence surveys to argue against a national public health strategy for gambling harms due to the relatively small percentage of the population in the ‘problem gambling’ category: 0.5% of adults in England in one estimate.^31^ Existing prevalence surveys have, however, well-recognised weaknesses due in part to a reliance on self-reports of a highly stigmatised addiction and challenges accessing high-risk groups:^32^ the Gambling Commission is, for this reason, developing a new survey instrument.

Advances in public health research reveal a continuum of diverse individual and social harms ranging from individual health impacts, which include suicide, to disruption to families, relationships and community cohesion.^33^ Seven percent of the UK population may be being harmed by someone else's gambling^31^ with a recent needs assessment by Greater Manchester Combined Authority estimating that 1 in 15 local residents are experiencing gambling harms.^34^ A Public Health England report, reviewed by the Office of Health Improvement and Disparities in 2023, estimated that the total national social cost of gambling in England is up to £1.27 billion.^31^ The bulk of this social cost may be concentrated at the low end and middle of the harms continuum because of the large numbers of ‘low-risk’ and ‘moderate’ gamblers.^35^

Taking these considerations into account, it becomes difficult to agree with the longstanding position of the BGC that, alongside universal safer gambling messaging, all that is required are targeted interventions for the minority of people who are severely harmed by their gambling.^7^ What is more, the stance jars when we consider that industry profits are disproportionally generated by consumers experiencing harm with one estimate, by Landman Economics, calculating that 20% of online profits accrues from ‘problem gamblers’.^36^ Extensive effort is expended, by the industry, to shape both situational factors (i.e. the supply and marketing of gambling products) and structural factors (i.e. the products themselves) to entice people in and keep them gambling once they start.^37^ Product innovations in online and machine gambling, purposefully designed to promote the establishment of repetitive gambling behaviours, appear to be particularly harmful.^38^ Natasha Dow Schüll, whose book ‘Addiction by Design’ is widely cited in the field, describes how these work through a self-contained environment, referred to as ‘The Zone’, in which players lose track of time and losses in continuous play.^39^ While regulation of the design and supply of these addictive products is urgently required, it is our view that industry manipulation of the situational and structural context of gambling presents opportunities for educational interventions to exploit.

## ‘Odds Are: They Win’

‘Odds Are: They Win’ is a social marketing campaign launched by the Greater Manchester Combined Authority (GMCA) in October 2022. One of the first of its kind in the UK, the campaign raises awareness of addictive products and harmful industry practices through various formats and media. The campaign was coproduced with a lived experience group called GaMHive, consisting of people with experience of gambling harms, providing advice on contents. GaMHive were presented with various design options after an initial consultation. Echoing academic criticism of safer gambling campaigns, GaMHive were critical of one set of images for resembling gambling advertisements too closely. On GaMHive's advice, a second set was developed that, while still retaining gambling imagery, featured bold and impactful statements on gambling harms and the gambling industry's role in facilitating them. Some GaMHive members believed that exposure to this stronger, industry-oriented focus may have made a difference to their own gambling behaviours:

*The fundamental message that I needed to hear at 16, 17 years old was that the gambling industry makes 14 billion a year. It doesn’t do that by making lots of winners. Ninety-nine percent of the customers lose. The other 1% get their accounts restricted or closed. This is the industry you’re up against (GaMHive member, personal communication)*

**Fig. 1:**
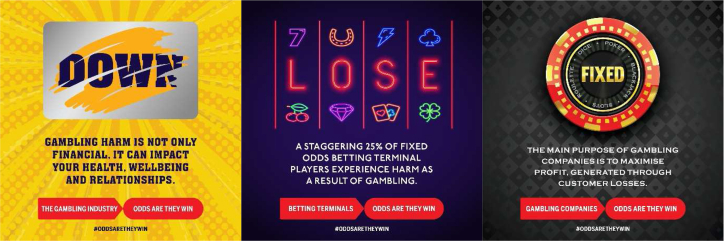
Examples of ‘Odds Are: They Win’ advertisements.

Example ‘Odds Are: They Win’ advertisements feature in Fig. 1. The DOWN advertisement features information on the diversity of gambling harms, as this is poorly understood among the public.^40^ Down denotes both financial loss and unhappiness. The scratch card image replaced an initial roulette wheel, as GaMHive advised the latter could be triggering. LOSE similarly denotes diverse financial and health-related losses. Information is presented on a particularly harmful product, the Fixed Odds Betting Terminal. The FIXED advertisement features a truism regarding the main purpose of the gambling industry with a view to countering marketing that presents operators as friends of people who gamble. These, and other advertisements were implemented over a three-month pilot period on social media and in diverse physical settings, including Greater Manchester's tram system and coffee shop chains. Service evaluation data revealed considerable reach, including an estimated +1.4 million people across the region.^41^

## A disruptive innovation?

We consider ‘Odds Are: They Win’ to be a potentially disruptive innovation in the gambling harms field, recognising that full ‘disruptive’ status would require individual responsibility–framed campaigns to be displaced more fully. The UK government recently announced an intention to replace industry-funded safer gambling messaging with independent and robustly evaluated public health messages.^42^ We propose that ‘Odds Are: They Win’ is evaluated, as part of this policy drive, to assess the potential of the tagline to raise awareness of the risks of gambling and associated harms. Interestingly, ‘Odds Are: They Win’ shares similarities with the Australian government's ‘Chances are you're about to lose’ tagline, which exhibited the strongest impact of 7 similar taglines on testing:^15^ i.e. they both target the information asymmetry that sees many people who gamble overstate the odds of winning.^43^ However, the direct reference to the gambling industry (i.e. ‘They’), along with the accompanying information on harmful products and practices, ensures ‘Odds Are: They Win’ breaks more conclusively with individual responsibility framings. The campaign is testament to how lived experience–led knowledge can disrupt established research paradigms.^44^

Key questions for ‘Odds Are: They Win's’ development pertain to future contents, media types, target groups, and delivery contexts. We would argue in favour of a preventative focus on low-risk and moderate-risk gamblers, as opposed to people who are experiencing severe harm: among the latter group, messages promoting sources of support may be more appropriate, although an important consideration is whether ‘Odds Are: They Win's’ industry-oriented focus has stigma-related benefits here. Among low-risk and moderate-risk users, with research suggesting that stereotypical notions of ‘the problem gambler’ can complicate individual problem recognition in a way similar to how ‘the alcoholic’ stereotype functions in the alcohol field,^45^ a research priority is to explore the responses of this group to ‘Odds Are: They Win’. Bypassing simplistic binaries that imply that harms are experienced by a small subset of people (as opposed to existing on a broad spectrum),^23^ ‘Odds Are: They Win’ could be more conducive to individual reflection and behaviour change than typical safer gambling campaigns.

‘Odds Are: They Win’ may also have broader relevance to how gambling harms are talked about in society and help counter strategic industry communications, which include efforts to pit consumers against public health actors.^46^ GMCA implemented ‘Odds Are: They Win’ in public settings to stimulate debate in the public sphere.^41^ This has potential implications for gambling narratives, social norms, and policy preferences. Public sphere discussion, prompted by ‘Odds Are: They Win’, could increase support for public health legislation for addressing gambling harms among people who do not gamble, or among people with positive gambling experiences in the past. ‘Odds Are: They Win's’ evaluation must, therefore, consider diverse criteria, target groups, and delivery settings. For, there may be positive externalities from the universal provision of information on harmful products and industry practices in markets characterised by major information asymmetry and social cost.

## Author statements

### Ethical approval

The study received ethical approval from the Institute of Health and Social Care Research Ethics Committee, London South Bank University [ETH2122-0114].

### Funding

This study was carried out at PHIRST South Bank, based at 10.13039/501100001261London South Bank University. It is funded by the 10.13039/100006662NIHR [Public Health Research (PHR) Programme NIHR131568/NIHR135398]. The views expressed are those of the author(s) and not necessarily those of the NIHR or the Department of Health and Social Care.

GMCA’s work (including funding for GamHive) is supported by the Gambling Commission as part of the National Strategy to Reduce Gambling Harms and funded by a regulatory settlement with an industry operator.

### Competing interests

TM, JG, EC, CJ, JE, JW and SS have not previously received research funding from the gambling industry, either directly or indirectly. TM, CJ, JW and SS are public health researchers who are new to gambling research. AM has previously received funding from GambleAware and acted as a paid consultant to the Safer Gambling Campaign Board. JG works for the charity Gambling with Lives that has previously received regulatory settlement money from the Gambling Commission for service development: JG’s participation in the research was funded via the NIHR grant. EC and JE’s roles in gambling harms reduction at the GMCA are funded by regulatory settlement money from the Gambling Commission as part of the National Strategy to Reduce Gambling Harms.
